# Association between underweight, serum albumin levels, and height loss in the Japanese male population: a retrospective study

**DOI:** 10.1186/s40101-024-00362-7

**Published:** 2024-05-27

**Authors:** Yuji Shimizu, Eiko Honda, Nagisa Sasaki, Midori Takada, Tomokatsu Yoshida, Kazushi Motomura

**Affiliations:** Epidemiology Section, Division of Public Health, Osaka Institute of Public Health, Osaka, Japan

**Keywords:** Albumin, Hyponutrition, BMI, Height loss

## Abstract

**Background:**

Previous study has shown that height loss (defined as the highest quartile of height loss per year) was inversely associated with serum albumin levels. Furthermore, comparatively healthy hyponutrition has been linked with being underweight; as such, underweight might be inversely associated with serum albumin levels and positively associated with height loss.

**Methods:**

To clarify the associations between serum albumin level, underweight status, and height loss, we conducted a retrospective study of 8,096 men over 4.0 years (median).

**Results:**

Serum albumin level at baseline was inversely associated with being underweight (body mass index [BMI]: < 18.5 kg/m^2^) at baseline and height loss. The known cardiovascular risk factor adjusted odds ratio (OR) and 95% confidence interval (CI) of underweight at baseline and of height loss for 1 standard deviation increment of serum albumin (0.28 g/dL) was 0.79 (0.70, 0.90) and 0.84 (0.80, 0.88). Underweight was also shown to be positively associated with height loss: with the reference of normal-low weight (BMI: 18.5–22.9 kg/m^2^), the adjusted OR (95% CI) was 1.60 (1.21, 2.10).

**Conclusion:**

Comparative healthy hyponutrition, which is related to low serum albumin levels and being underweight, is a significant risk factor for height loss among Japanese men. These results help to clarify the mechanisms underlying height loss.

## Introduction

A previous study on Japanese general workers identified a significant inverse association between serum albumin level and height loss, which was defined as the highest quartile of height loss per year [1]. However, the mechanism underlying this inverse association has not yet been clarified.

Another study of Japanese general workers showed a significant positive association between eating speed and height loss, defined as being in the highest quintile of height decrease per year among non-overweight individuals (body mass index [BMI] below 25.0 kg/m^2^). In this study, high BMI (≥ 25.0 kg/m^2^) was found to be positively associated with eating speed and height loss [2].

Since being underweight, which is related to hyponutrition, has been linked to reduced serum concentrations of albumin, these studies evoke and contradict these findings.

Hyponutrition, which is associated with low serum albumin levels, is a risk factor for height loss [1], whereas hypernutrition, which is related to high BMI, is also positively associated with height loss [2].

Height loss starting in middle age may be associated with all-cause and cardiovascular disease-related mortality [3]. Endothelial repair activity evaluated by the number of circulating endothelial progenitor cell (CD34-positive cell) has also been reported to be inversely associated with height loss [4]. Low levels of circulating CD34-positive cell are associated with an increased risk of cardiovascular disease [5, 6].

Therefore, clarifying the associations between serum albumin, underweight, and height loss could aid in developing a novel strategy for cardiovascular prevention as the target population for preventing cardiovascular disease.

## Materials and methods

### Study population

The study population comprised 14,494 residents of Yao city, an urban community in western Japan, aged 40–73 years, who underwent an annual medical check-up from 2014 to 2021. The first checkup was considered the baseline. Participants without a drinking habits data (*n* = 14), or without serum data (*n* = 2,554), were excluded. We further excluded 3,049 participants who did not undergo an annual health checkup during the follow-up period (2015–2022). Participants who were followed up for less than 1 year (*n* = 781) were also excluded from present study. The remaining participant, 8,096 men aged 63.0 years ± 9.0 years (range, 40–73 years), were enrolled in the study. The mean follow-up period (standard deviation [SD]) was 3.4 (1.2) years.

This study was approved by the ethics committee of Osaka Institute of Public Health (project registration code: 2311–02). All procedures involving human participants were performed in accordance with the ethical standards of the Ethics Committee of Osaka Institute of Public Health and the 1964 Declaration of Helsinki and its amendments. Informed consent for this study was obtained from all participants for study population using the opt-out method, with descriptions of the study presented on the institution’s website (https://www.iph.osaka.jp/s004/099/2311-02.pdf) (accessed on 12th January 2024). All analyses for this study were conducted using deidentified data, and the authors completing the analysis did not have access to data with identifiers. For research purposes data were extracted 26th December 2023.

### Data collection and laboratory measurements

The data used in this study were statutory reporting data of annual health checkups performed in Yao City. Because these annual health check-ups are supported by the Japanese government, the methods used for data collection and laboratory measurements are nationally standardized.

### Baseline data

The baseline period of this study was from 2014 to 2021. BMI was calculated as weight divided by height in meters squared (kg/m^2^). The resting blood pressure was measured twice. Fasting blood samples were collected from all patients. Albumin, triglyceride (TG), high-density lipoprotein cholesterol (HDLc), low-density lipoprotein cholesterol (LDLc), and Hemoglobin A1c (HbA1c) levels were also measured.

BMI categories were defined as follows: underweight, BMI < 18.5 kg/m^2^; normal-low weight, BMI: 18.5–22.9 kg/m^2^; normal-high weight, BMI:23.0–24.9 kg/m^2^; overweight, BMI: 25.0–29.9 kg/m^2^ and obesity, BMI ≥ 30 kg/m^2^. Hypertension was defined as systolic blood pressure ≥ 140 mmHg and diastolic blood pressure ≥ 90 mmHg. Dyslipidemia was defined as TG ≥ 150 mg/dL, LDLc ≥ 140 mg/dL, HDLc < 40 mg/dL. Higher HbA1c was defined as HbA1c (5.6–6.4%) and high HbA1c as HbA1c (≥ 6.5%).

### Endpoint data

The endpoint of this study was from 2015 to 2022. During baseline evaluations, the height (cm) was measured. Decreases in height per year (cm/year) were calculated using height measurements at baseline and at the endpoint. Height loss was defined as being in the highest quartile of annual height decrease (decreased by 0.23 cm/year), as in previous studies [1, 4].

### Statistical analysis

The characteristics of the study population were calculated according to serum albumin levels and BMI categories. Age was expressed as means ± SD. Obesity, overweight, normal-high weight, normal-low weight, underweight, hypertension, daily drinker, non-drinker, smoker, higher HbA1c, high HbA1c, and dyslipidemia are also shown as percentages. Significant differences were evaluated using the Jonckheere-Terpstra trend test for continuous variables and the Cochran-Armitage trend test for proportions.

Logistic regression was applied to calculate the odds ratios (ORs) and 95% confidence intervals (CIs) of being underweight and incidence of height loss for serum albumin. The ORs and 95% CIs of height loss for the BMI categories were calculated using a logistic regression model.

Height loss is reportedly associated with cardiovascular mortality [3], and may also be influenced by endothelial repair [4]. Therefore, the cardiovascular risk factors may have acted as confounders in this study. High BMI (≥ 25.0 kg/m^2^) [2], hypertension [7], higher HbA1c (5.6–6.5%) [8] were reported to be positively associated with height loss. Diabetes, which is related to high HbA1c (≥ 6.5%), is an established risk factor for intervertebral disk degeneration [9]. Two different approaches were used to adjust for the confounding factors. First, we adjusted for age (age-adjusted model). In the multivariate model, the adjustment factors were dependent on the analysis. For the analysis between serum albumin and underweight status, we included several other potential confounding factors, namely age, obesity (yes, no), overweight (yes, no), hypertension (yes, no), daily drinker (yes, no), non-drinkers (yes, no), smoker (yes, no), higher HbA1c (yes, no), high HbA1c (yes, no), and dyslipidemia (yes, no). For the analysis of serum albumin levels and height loss, we adjusted for age, obesity (yes, no), overweight (yes, no), underweight (yes, no), hypertension (yes, no), daily drinker (yes, no), non-drinker (yes, no), smoker (yes, no), higher HbA1c (yes, no), high HbA1c (yes, no), and dyslipidemia (yes, no). To assess the association between BMI categories (underweight, normal-low weight, normal-high weight, overweight, and obesity) and height loss, age, hypertension (yes or no), daily drinker (yes or no), non-drinker (yes or no), smoker (yes or no), higher HbA1c (yes or no), high HbA1c (yes or no), and dyslipidemia (yes or no) were adjusted for.

Statistical significance was set at *p* < 0.05. All statistical analyses were performed using SAS for Windows (version 9.4; SAS Inc., Cary, NC, USA).

## Results

### Characteristics of the study population in relation to serum albumin levels

Table 1 shows the characteristics of the study population according to serum albumin levels. Overweight, hypertension, higher HbA1c, high HbA1c, and dyslipidemia were significantly positively associated with serum albumin levels, while age, normal-low weight, daily drinker, and smoker were significantly inversely associated with serum albumin levels.

**Table 1:** Characteristics of the study population in relation to serum albumin levels

	Serum albumin				*P* for trend
	Quartile 1 (low)	Quartile 2	Quartile 3	Quartile 4 (high)	
No. of participants	1,642	2,216	2,259	1,979	
Age, year	65.5 ± 6.9	64.0 ± 8.0	62.5 ± 9.1	60.2 ± 10.3	< 0.001
Obesity, %	3.8	3.7	4.1	5.4	0.163
Overweight, %	23.7	28.5	30.4	31.7	0.005
Normal high weight, %	24.9	26.1	26.6	27.3	0.081
Normal low weight, %	42.0	38.3	36.6	33.5	0.003
Underweight, %	5.6	3.3	2.3	2.2	0.538
Hypertension, %	26.9	28.9	28.2	32.7	< 0.001
Daily drinker, %	53.8	48.4	46.5	40.8	< 0.001
Non-drinker, %	29.2	33.8	33.8	36.2	0.690
Smoker, %	31.7	27.7	27.0	25.3	0.002
Higher HbA1c, %	43.6	46.5	45.6	46.4	<0.001
High HbA1c, %	11.9	11.0	12.0	14.1	0.0032
Dyslipidemia, %	40.7	48.6	51.3	55.8	< 0.001

Obesity: BMI(≥30.0 kg/m^2^). Overweight: BMI (25.0–29.9 kg/m2). Normal high weight: BMI (23.0–24.9 kg/m^2^). Normal low weight: BMI (18.5–22.9 kg/m^2^). Underweight: BMI (<18.5 kg/m^2^ ). Hypertension: systolic blood pressure≥140 mmHg and/or diastolic blood pressure≥90 mmHg. Higher HbA1c: 5.6–6.4%. High HbA1c: ≥6.5%. Serum albumin levels: < 4.2 g/dL for quartile 1 (low), 4.2–4.3 g/dL for quartile 2, 4.4–4.5 g/dL for quartile 3, and ≥ 4.6 g/dL for quartile 4 (high)

*BMI* body mass index

### Characteristics of the study population in relation to BMI categories

The characteristics of the study population according to the BMI categories are presented in Table 2. Hypertension, higher HbA1c, high HbA1c, and dyslipidemia were significantly positively associated with BMI categories, whereas non-drinkers, and smokers showed a significant inverse association with BMI categories. An inverse U-shaped association was observed between daily drinker and the BMI categories.

**Table 2:** Characteristics of the study population in relation to BMI categories

	BMI categories					*P* for trend
	Underweight	Normal low	Normal high	Overweight	Obesity	
No. of participants	262	3027	2129	2334	344	
Age, year	63.5 ± 9.0	63.5 ± 8.9	63.8 ± 8.3	62.4 ± 9.1	57.1 ± 10.5	0.9901
Hypertension, %	14.9	25.3	28.3	34.1	47.1	< 0.001
Daily drinker, %	43.1	48.4	49.0	45.2	39.5	<0.001
Non-drinker, %	43.1	32.3	32.0	35.2	33.7	<0.001
Smoker, %	39.3	29.9	26.2	25.2	26.5	0.037
Higher HbA1c, %	33.2	41.7	46.6	50.8	49.1	< 0.001
High HbA1c, %	8.0	9.4	11.6	14.9	25.3	< 0.001
Dyslipidemia, %	19.8	40.8	51.5	59.9	65.1	< 0.001

Hypertension: systolic blood pressure≥140 mmHg and/or diastolic blood pressure≥90 mmHg. Higher HbA1c: 5.6–6.4%. High HbA1c: ≥6.5%. BMI categories: < 18.5 kg/m^2^ for underweight, 18.5–22.9 kg/m^2^ for normal low, 23.0–24.9 kg/m^2^ for normal high, 25.0–29.9 kg/m^2^ for overweight, and ≥ 30.0 kg/m^2^ for obesity

### Association between serum albumin and underweight

Independent of known potential confounders, a significant inverse association between serum albumin levels and underweight status was also observed (Table 3).

**Table 3:** Association between serum albumin and underweight status

	Serum albumin				*P* for trend	1 SD increment of serum albumin
	Quartile 1 (low)	Quartile 2	Quartile 3	Quartile 4 (high)		
No. of participants	1,642	2,216	2,259	1,979		
No. of underweight, n (%)	92 (5.6)	74 (3.3)	52 (2.3)	44 (2.2)		
Age-adjusted model	Reference	0.58 (0.42, 0.79)	0.39 (0.28, 0.56)	0.39 (0.26, 0.55)	< 0.001	0.69 (0.61, 0.78)
Multivariable model	Reference	0.70 (0.50, 0.96)	0.50 (0.35, 0.71)	0.55 (0.37, 0.81)	< 0.001	0.79 (0.70, 0.90)

Multivariable model: Adjusted for age, obesity, overweight, hypertension, daily drinker, non-drinker, smoker, higher HbA1c, high HbA1c. *SD* standard deviation. 1 SD is 0.28 g/dL. Serum albumin levels: < 4.2 g/dL for quartile 1 (low), 4.2–4.3 g/dL for quartile 2, 4.4–4.5 g/dL for quartile 3, and ≥ 4.6 g/dL for quartile 4 (high)

### Association between serum albumin and height loss

Independent of known potential confounders, a significant inverse association was observed between serum albumin level and height loss (Table 4).

**Table 4:** Association between serum albumin and height loss

	Serum albumin				*P* for trend	1 SD increment of serum albumin
	Quartile 1 (low)	Quartile 2	Quartile 3	Quartile 4 (high)		
No. of participants	1,642	2,216	2,259	1,979		
No. of height loss, n (%)	520 (31.7)	590 (26.6)	522 (23.1)	392 (19.8)		
Age-adjusted model	Reference	0.82 (0.71, 0.94)	0.70 (0.61, 0.81)	0.62 (0.53, 0.72)	< 0.001	0.83 (0.79, 0.88)
Multivariable model	Reference	0.83 (0.72, 0.96)	0.72 (0.62, 0.83)	0.63 (0.54, 0.74)	< 0.001	0.84 (0.80, 0.88)

Multivariable model: Adjusted for age, underweight, overweight, obesity, hypertension, daily drinker, non-drinker, smoker, higher HbA1c, high HbA1c, dyslipidemia. *SD* standard deviation. 1 SD is 0.28 g/dL. Serum albumin levels: < 4.2 g/dL for quartile 1 (low), 4.2–4.3 g/dL for quartile 2, 4.4–4.5 g/dL for quartile 3, and ≥ 4.6 g/dL for quartile 4 (high)

### Association between BMI categories and height loss

Independent of known potential confounders, in the normal-low weight reference group, underweight patients showed significantly higher ORs for height loss. In the reference group with a normal-low weight, obesity showed significantly higher ORs for height loss. This association remained significant even after adjusting for known confounders (Table 5).

**Table 5:** Association between categories of body mass index (BMI) and height loss

	**BMI categories**								
	Underweight		Normal low	Normal high		Overweight		Obesity	
		p			p		p		p
No. of participants	262		3,027	2,129		2,334		344	
No. of height loss, n (%)	91 (34.7)		747 (24.7)	522 (24.5)		574 (24.6)		90 (26.2)	
Age-adjusted model	1.64 (1.26, 2.14)	< 0.001	Reference	0.98 (0.86, 1.12)	0.777	1.04 (0.96, 1.18)	0.587	1.36 (1.05, 1.77)	0.021
Multivariable model	1.60 (1.21 2.10)	< 0.001	Reference	1.00 (0.88, 1.14)	0.972	1.06 (0.93, 1.21)	0.389	1.37 (1.05, 1.79)	0.019

Multivariable model: Adjusted for age, hypertension, daily drinker, non-drinker, smoker, higher HbA1c, high HbA1c, dyslipidemia. BMI categories: < 18.5 kg/m^2^ for underweight, 18.5–22.9 kg/m^2^ for normal low, 23.0–24.9 kg/m^2^ for normal high, 25.0–29.9 kg/m^2^ for overweight, and ≥ 30.0 kg/m^2^ for obesity

### Associations between height loss, defined as being in the highest quintile of annual height decrease

In addition, we performed the same analyses with height loss, defined as being in the highest quintile of the annual height decrease. The association between serum albumin levels and height loss, and the association between BMI categories and height loss were also calculated. Overall, the age-adjusted and multivariable ORs (95% CIs) of height loss for a 1 SD increment of serum albumin were 0.83 (0.79, 0.88) and 0.84 (0.80, 0.89). In the normal-low weight reference group, the age-adjusted and multivariable ORs (95% CIs) of height loss for underweight status were 1.88 (1.43, 2.49) and 1.85 (1.40, 2.46), respectively. The reference group was normal-low weight. the age adjusted ORs (95% CIs) of height loss for normal-high weight, overweight and obesity were 0.96 (0.83, 1.01), 1.07 (0.93, 1.22), and 1.36 (1.02, 1.80). The corresponding values in the multivariable model were 1.00 (0.88, 1.14), 1.06 (0.93, 1.21) and 1.37 (1.05, 1.79), respectively.

## Discussion

In this study, being underweight, which is inversely associated with serum albumin levels, was found to be positively associated with height loss among Japanese men. A previous study of 8,982 Japanese worker aged 40 to 74 years showed significant positive association between high BMI (≥ 25.0 kg/m^2^) and height loss (defined as being in the highest quintile of height decrease per year) [2]. This finding is partly consistent with the present results. In the present study, even overweight (25.0–29.9 kg/m^2^) status did not show any significant associated with the risk of height loss.

**Fig. 1:**
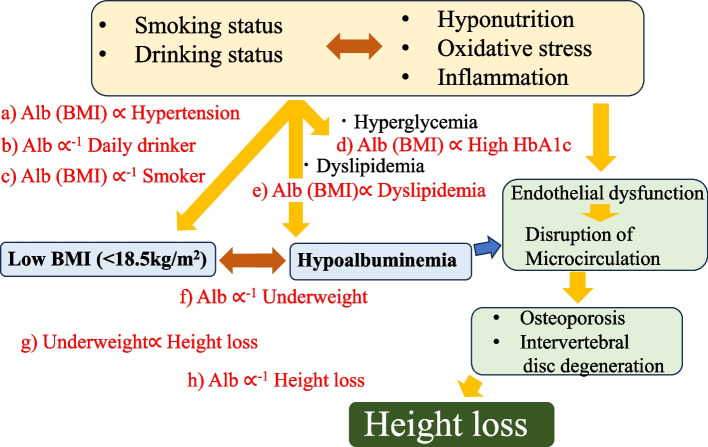
Possible mechanism underlying height loss. *Alb* albumin. Relations in red (a ~ h) were observed in this study

The present study found further evidence that being underweight (< 18.5 kg/m^2^) is a significant risk factor for height loss among Japanese men. However, the mechanisms underlying the association between underweight and height loss remain unknown. Figure 1 shows the potential mechanisms underlying these results.

Compression fractures of the spine and narrowed intervertebral space are two major causes of height loss among adults. Furthermore, osteoporosis causes compression fractures and vertebral disc degeneration causes narrowing of the intervertebral space.

Disruption of the microcirculation has been reported as being associated with osteoporosis [10]. Disturbance of blood flow to the intervertebral discs may also play a crucial role in the development of lumbar disc degeneration [11].

CD34-positive cells, which play an important role in endothelial repair [12] contribute to the maintenance of microcirculation by promoting angiogenesis [13] and neovascularization [14]. The number of circulating CD34-postive cells can be used as a marker of endothelial repair activity [15]. As circulating CD34-positive cells have been reported to be inversely associated with height loss [4], blood flow disturbance, which is related to a shortage of endothelial repair, may cause height loss in the general population.

Because serum albumin influences the migration of CXCR4-expressing primary CD34 + hematopoietic progenitor cells [16], low levels of serum albumin may be associated with height loss [1], indicating the disadvantage of activating endothelial repair. In the present study, serum albumin was found to be inversely associated with height loss (Table 4) (Fig. 1h) and underweight status (Table 3) (Fig. 1f). This may be because underweight participants might have a disadvantage in activating endothelial repair, which might cause height loss. Therefore, underweight status was positively associated with height loss (Table 5) (Fig. 1g), partly because of the presence of lower levels of albumin.

In addition to endothelial repair activity, serum albumin contributes to extracellular antioxidant defenses [17]. as such, higher serum albumin levels may have higher antioxidant activity than lower serum albumin levels. Smoker, hypertension, dyslipidemia higher HbA1c and high HbA1c showed essentially the same association with serum albumin levels and BMI categories in the present study. Smoker showed a significant inverse association with serum albumin levels (Table 1) and BMI categories (Table 2) (Fig. 1c). Hypertension, dyslipidemia, higher HbA1c, and high HbA1c levels were significantly positively associated with serum albumin levels (Table 1) and BMI categories (Table 2) (Fig. 1a, d, e). These associations may be explained by nutritional status.

Conversely, in daily drinkers, serum albumin levels showed a linear inverse association (Table 1) (Fig. 1b), whereas BMI categories showed an inverse U-shaped association (Table 2). Similarly, in the present study, serum albumin levels showed a linear inverse association with height loss (Table 4), whereas the BMI categories showed an inverse U-shaped association with height loss (Table 5). Platelets contribute to endothelial repair in conjunction with circulating CD34-positive cell [18]. Ethanol directly attenuates platelet activation [19] and influences on endothelial health [20]. Therefore, differences in the distribution of daily drinkers may partly explain the difference between serum albumin and BMI categories as risk factors for height loss. Further investigations on platelet activity are necessary.

Postural changes during the observation period may also have played an important role in the present associations. There are bidirectional relationships between postural changes and a compression fracture of the spine, and between postural change and a narrowed intervertebral space due to intervertebral disc degeneration. Compression fractures of the spine and intervertebral disc degeneration, which can cause postural changes, can also be induced by decreased thoracic extensor muscle strength, which is related to postural changes [21, 22]. Therefore, postural changes might act as a mediator of the association between the serum albumin level and height loss, and the association between BMI categories and height loss.

The primary clinically relevant finding of the present study is that both obesity and underweight status increase the risk of height loss among Japanese men. Serum albumin partly acts as a marker of height loss, which is related to underweight status.

The limitations of this study warrant further consideration. Firstly, we only investigated associations in men. Although essentially the same associations were observed for women, the goodness of fit test evaluated by the Hosmer–Lemeshow test was not validated for the analyses among women. As menopausal status may influence height loss, a more detailed study including information on menopausal status may be necessary for women. Although the highest quartile of height decrease per year was defined as height loss in the present study, an efficient cut-off point to define height loss has not been established. However, additional analyses that defined height loss using the highest quintile of height decrease per year showed essentially the same associations.

## Conclusion

In conclusion, the present study showed that being underweight, which is inversely associated with serum albumin levels, was positively associated with height loss among Japanese men. These findings could help to clarify the mechanisms underlying height loss in Japanese men.

## Data Availability

The datasets generated and/or analyzed during the current study are not publicly available due to ethical considerations. Qualified researchers may apply for access a minimal dataset by contacting data management staff at epiana@iph.osaka.jp. Information regarding data requests is also available at https://www.iph.osaka.jp/s016/000/index.html (accessed on 12the January 2024).

## Abbreviations

BMI: Body mass index
OR: Odds ratio
CI: Confidence interval
SD: Standard deviation
TG: Triglycerides
HDLc: High-density lipoprotein cholesterol
LDLc: Low-density lipoprotein cholesterol
HbA1c: Hemoglobin A1c
